# Additional germline findings from a tumor profiling program

**DOI:** 10.1186/s12920-018-0383-5

**Published:** 2018-08-09

**Authors:** Neda Stjepanovic, Tracy L. Stockley, Philippe L. Bedard, Jeanna M. McCuaig, Melyssa Aronson, Spring Holter, Kara Semotiuk, Natasha B. Leighl, Raymond Jang, Monika K. Krzyzanowska, Amit M. Oza, Abha Gupta, Christine Elser, Lailah Ahmed, Lisa Wang, Suzanne Kamel-Reid, Lillian L. Siu, Raymond H. Kim

**Affiliations:** 1 Division of Medical Oncology and Hematology, Princess Margaret Cancer Centre, 610 University Ave, Toronto, ON M5G 2M9 Canada; 2 Cancer Genomics Program, Princess Margaret Cancer Centre, 610 University Ave, Toronto, ON M5G 2M9 Canada; 3 Department of Clinical Laboratory Genetics & Department of Laboratory Medicine and Pathobiology, University of Toronto, 610 University Ave, Toronto, ON M5G 2M9 Canada; 4 Department of Molecular Genetics, University of Toronto, 610 University Ave, Toronto, ON M5G 2M9 Canada; 5 Zane Cohen Centre for Digestive Diseases, Mount Sinai Hospital, 60 Murray St, Toronto, ON M5T 3L9 Canada; 6 Department of Biostatistics, Princess Margaret Cancer Centre, 610 University Ave, Toronto, ON M5G 2M9 Canada

**Keywords:** Germline mutation, Neoplasms/genetics, Neoplastic syndromes, Hereditary Cancer, Incidental findings, Secondary findings, Next generation sequencing

## Abstract

**Background:**

Matched tumor-normal sequencing, applied in precision cancer medicine, can identify unidentified germline Medically Actionable Variants (gMAVS) in cancer predisposition genes. We report patient preferences for the return of additional germline results, and describe various gMAV scenarios delivered through a clinical genetics service.

**Methods:**

Tumor profiling was offered to 1960 advanced cancer patients, of which 1556 underwent tumor-normal sequencing with multigene hotspot panels containing 20 cancer predisposition genes. All patients were provided with an IRB-approved consent for return of additional gMAVs.

**Results:**

Of the whole cohort 94% of patients consented to be informed of additional germline results and 5% declined, with no statistically significant differences based on age, sex, race or prior genetic testing. Eight patients were found to have gMAVs in a cancer predisposition gene. Five had previously unidentified gMAVs: three in *TP53* (only one fulfilled Chompret’s Revised criteria for Li-Fraumeni Syndrome), one in *SMARCB1* in the absence of schwannomatosis features and one a *TP53* variant at low allele frequency suggesting an acquired event in blood.

**Conclusion:**

Interest in germline findings is high among patients who undergo tumor profiling. Disclosure of previously unidentified gMAVs present multiple challenges, thus supporting the involvement of a clinical genetics service in all tumor profiling programs.

**Electronic supplementary material:**

The online version of this article (10.1186/s12920-018-0383-5) contains supplementary material, which is available to authorized users.

## Background

Tumor profiling through next generation sequencing (NGS) has facilitated precision cancer therapies by identification of actionable tumor variants to guide cancer patient management [1]. Genetic analysis of tumor tissue can detect both acquired (somatic) aberrations found exclusively in the cancer cells, and inherited (germline, constitutional) variants. Often in molecular profiling of tumors, germline DNA from normal tissue is also tested to aid in filtering tumor-specific events by identification and subtraction of germline variants [2]. However the analysis of germline DNA may identify pathogenic germline variants in cancer predisposition genes included in NGS molecular profiling panels [3]. The American Society of Clinical Oncology (ASCO) and the Clinical Sequencing Exploratory Research (CSER) Consortium Tumor Working Group, support the communication of medically relevant secondary or incidental germline findings from tumor profiling programs based on patient preferences [4, 5]. CSER defines secondary findings as “results that are unrelated to the diagnostic question, but are systematically sought and analyzed, while incidental findings are not sought out, but identified nonetheless” [6]. Recently, these findings have been collectively referred to as “additional findings” based on patients’ preferences [7]. A number of NGS tumor profiling programs have reported additional germline findings in actionable cancer predisposition genes with the frequency ranging between 4.3 and 17.5% of patients tested [8–11]. While studies designed to actively seek secondary gMAVs require considerable amount of analysis and resources, studies designed not to actively seek gMAV may also encounter additional findings incidentally. Although at a lower frequency, mechanisms to incorporate such additional findings into the clinical care of these patients should be considered. Tumor profiling programs may also provide a new avenue to identify individuals with a cancer predisposition syndrome with implications on their clinical management and families.

The Princess Margaret Cancer Centre completed accrual of two tumor profiling studies, the Integrated Molecular Profiling in Advanced Cancers Trial (IMPACT) and Community Oncology Molecular Profiling in Advanced Cancers Trial (COMPACT). Two targeted NGS panels of 48–50 genes were analyzed to inform precision cancer therapies in advanced cancer patients through paired tumor-germline sequencing [12]. Peripheral blood lymphocytes (PBL) were selected as representative of normal tissue to identify germline variants to aid in identification of tumor-specific variants. Although the variant analysis was not designed to detect all germline variants in cancer predisposition genes in the tested panels, the potential of detecting germline medically actionable variants (gMAVs) incidentally was recognized. Information about gMAVs was offered to the patients and disclosed only to those who provided consent. Here, we describe patient preferences in the return of additional gMAVs in cancer predisposition genes detected through tumor profiling, the types of variants detected and considerations in the interpretation and disclosure of the findings.

## Methods

### Patient cohort

**Fig. 1:**
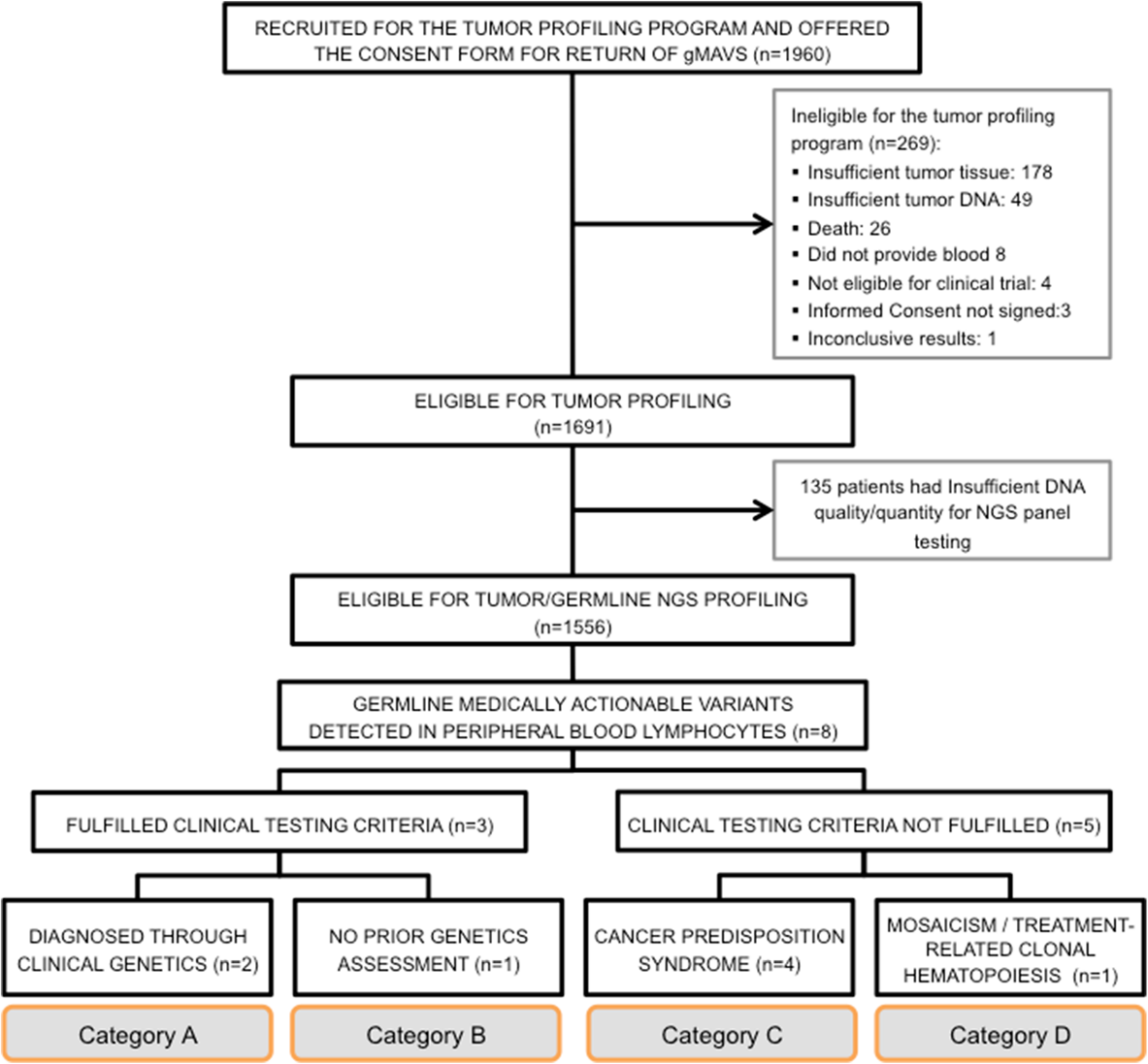
Patient recruitment and additional germline findings. *gMAV* Germline Medically Actionable Variant, *NGS* Next generation sequencing

The patient cohort consisted of advanced cancer patients who were candidates for clinical trials with targeted therapies and enrolled in the tumor profiling programs IMPACT or COMPACT (NCT01505400) (Fig. 1) [12]. Patients were age ≥ 18 years, with Eastern Cooperative Oncology Group (ECOG) performance status ≤1, had available formalin-fixed embedded archival tumor tissue and provided a blood sample to represent the germline DNA from PBL. At study registration all participants were asked to provide information regarding prior germline testing. Written informed consent for tumor profiling and germline co-analysis was obtained from all participants. An additional University Health Network Research Ethics Board-approved consent form for return of gMAVs was offered to the participants from June 2013 for IMPACT and January 2014 for COMPACT, until the closure of both trials in December 2015. Participants interested in the return of gMAV results were asked to identify a delegate (preferably biologic relative), who could receive the results on their behalf if required. Demographic and clinical data were extracted from prospectively maintained databases and medical records.

### Genetic analysis

DNA extraction and molecular analysis on PBLs or tumor FFPE tissue was performed as previously described [12]. NGS molecular test methods used included one of the following targeted amplicon cancer panels, designed to detect hotspot variants in regions of selected genes with known utility in somatic cancers: 1) TruSeq Amplicon Cancer Panel (TSACP; Illumina, San Diego, CA) sequenced on the MiSeq benchtop sequencer (Illumina), which included hotspot regions of 48 genes. (Additional file 1: Table S1) Sequence alignment and base calling used MiSeq Reporter (Illumina), followed by variant calling using NextGENe v.2.3.1 software (SoftGenetics, State College, PA) and data review using the Integrative Genomics Viewer (IGV, Broad Institute); or 2) Ion AmpliSeq Cancer Panel (ASCP; ThermoFisher Scientific, Waltham, MA) sequenced on the Ion Proton benchtop sequencer (ThermoFisher Scientific), which included hotspot regions of 50 genes (Additional file 1: Table S2) Sequence alignment and base calling was performed by Torrent Suite software (ThermoFisher Scientific) and analysis using NextGENe v.2.3.1 and IGV software.

Somatic variants identified met laboratory-defined thresholds of > 500× read coverage and allele frequency of > 10%. Recurrent mutations between 400-500X coverage or 5–10% allele fraction were reported if they were verified by an orthogonal molecular method. Three genes with read depth consistently falling below 500× on TSACP (*GNAS*, *HRAS*, *CDKN2A*) were not included in the data analysis.

Selected targeted hotspot regions (i.e. partial gene regions, not full gene/full exon sequences) of 20 genes which also have inherited cancer risk were included in the panels (Additional file 1: Table S1 and S2). For samples with insufficient DNA quality or quantity for either NGS panel, a custom multiplex genotyping assay was performed only on tumor tissue [12]. Tumor profiling with NGS methods was only performed when germline DNA was available. Germline and tumor samples from the same patient were tested using the same methods and analyses, and variants identified in tumor DNA were compared to variants identified in germline DNA to identify tumor-specific events. All NGS analyses used hg19, NCBI Build 37, as reference genome. All testing was performed in a laboratory accredited by the College of American Pathologists and certified to meet Clinical Laboratory Improvement Amendments.

### Determination of germline variants in cancer predisposition genes

Among the genes with targeted partial hotspot regions evaluated on the TSACP and ASCP NGS panels, 20 genes were related to cancer predisposition syndromes (Additional file 1: Tables S1–S3). Any germline variants detected in the select hotspot targeted regions of the cancer predisposition genes analyzed were investigated in online mutation databases (ClinVar, HGMD, IARC TP53, BIC), population variant databases (dbSNP, ExAC, 1000 Genomes) and relevant literature, and classified as pathogenic, likely pathogenic, uncertain significance, likely benign or benign using the variant assessment guidelines as specified by the American College of Medical Genetics [13]. The variant analysis approach was not specifically designed to systematically detect all germline variants as the focus of the data analysis was on primary detection of somatic acquired mutations. However, gMAVs were still identified incidentally. The gMAVs were defined as those germline variants which were pathogenic or likely pathogenic, were associated with a cancer predisposition syndrome and could have a clinical impact on the patient and/or prompt genetic testing in family members. gMAVs were considered as non-constitutional (mosaic or somatic event in PBL) when the allelic frequency of the variant in germline DNA was less than 25–30% based on validation data of the two NGS panels.

### Return of germline medically actionable variants in cancer predisposition genes

A “Genomics Tumor Board” was developed which included medical oncologists, clinical molecular laboratory geneticists, genetic counsellors and a medical geneticist. All pathogenic or likely pathogenic variants from germline DNA analysis, as well as variants of conflicting interpretation for cancer predisposition syndromes were reviewed in conjunction with the personal and family history to determine clinical significance and potential management steps. Each case was discussed independently to determine whether germline results would be returned to patient or their delegate. If a cancer predisposition syndrome was previously identified through standard clinical routes, no further action was taken. For potentially newly uncovered gMAVs in cancer predisposition genes, patients who consented to return of additional findings or their delegate were contacted by the clinical genetics service, which comprised of a medical geneticist and genetic counsellor. Confirmation of the germline results on a new sample in an accredited clinical laboratory was required prior to being incorporated into the patient’s medical record. Surveillance recommendations and familial cascade testing was conducted through standard clinical genetics routes (Additional file 2: Figure S1).

### Statistical analysis

Descriptive statistics were used to summarize patient demographics (age, gender, race, tumor type, ECOG and prior germline testing). Comparisons between patients who consented for return of additional gMAVs and those who did not, were performed using t-test for age and Chi-Square test for gender, race, tumor type, ECOG and prior genetic testing. Differences with *p*-values of < 0.05 were considered statistically significant. All statistical analyses were conducted in SAS, version 9.4.

## Results

### Consenting rates and patients’ preferences

**Fig. 2:**
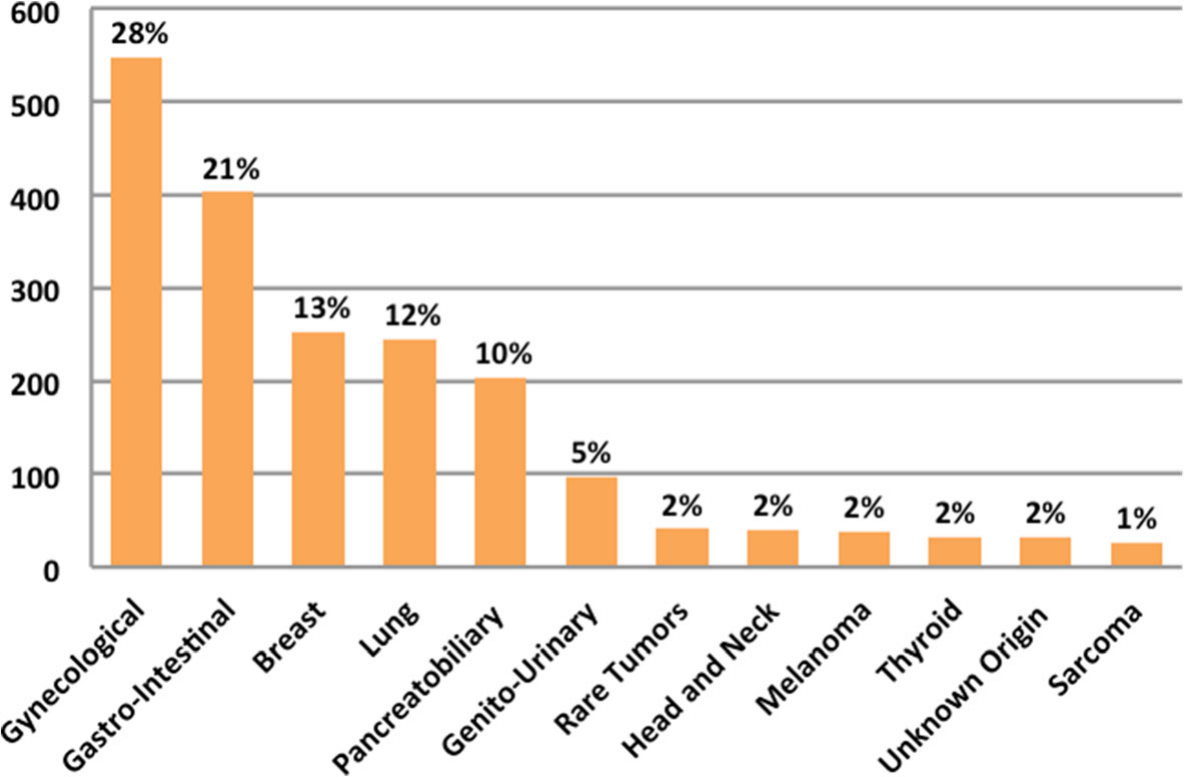
Patients’ characteristics – disease site

A total of 1960 patients with a variety of malignancies were consented for IMPACT and COMPACT. The median age at enrollment to both studies was 58 years (range 18–89 years) and 67% of the population was female. Other relevant clinical characteristics are depicted in Fig. 2 and Additional file 1: Table S4. Of note, 18% (361/1960) patients did report already having clinical germline genetic testing which is consistent with the referral rates in our centre [14]. In the consent form 1844 (94%) agreed to the return of additional pathogenic germline results, 103 (5%) declined and 13 (1%) improperly filled the section regarding additional findings. There was no statistically significant difference by age, sex, race or prior genetic testing among the patients who consented for return of germline results and those who declined (Table 1).

**Table 1:** Patients’ characteristics and acceptance of the return of secondary germline Medically Actionable Variants

	Accepted *n* = 1844	Declined *n* = 103	*p-value* ^†^
Age– years (mean)	57.6	57.6	> 0.95
Gender - n			*p-value* ^‡^
Female	1235 (67%)	71 (69%)	> 0.68
Male	609 (33%)	32 (31%)	
Race - n			
White	1003 (54%)	56 (54%)	> 0.95
Asian	207 (11%)	16 (16%)	
Black	35 (2%)	1 (1%)	
Mixed	14 (1%)	0 (0%)	
Unknown	585 (32%)	30 (29%)	
Cancer site - n			
Gynecological	518 (28%)	25 (24%)	0.015
Gastrointestinal	382 (21%)	19 (18%)	
Breast	233 (13%)	18 (17%)	
Lung	227 (12%)	17 (17%)	
Pancreas	194 (11%)	8 (8%)	
Sarcoma	21 (1%)	5 (5%)	
Others	269 (15%)	11(11%)	
Prior genetic testing - n			
Yes	345 (19%)	15 (15%)	0.292
No	1499 (81%)	88 (85%)	
ECOG - n			
0	760 (41%)	37 (36%)	0.288
1	1084 (59%)	66 (64%)	

*ECOG* The Eastern Cooperative Oncology Group, *n* number. Values are expressed as mean (+/− standard deviation), except otherwise stated. ^†*.*^ *T-d’Student test;* ^‡*.*^ *Chi square test*

### Variants detected through germline DNA analysis

Samples from 1556 patients were tested with NGS panels, and eight patients were found to have gMAVs in cancer predisposition genes Fig. 1 and Table 2.

**Table 2:** Characteristics of patients with germline Medically Actionable Variants

Pt	Cat	Sex	Cancer (Age at diagnosis)	HCS	Variant in PBL (AF)
1	A	F	Desmoid tumor (32), Rectal cuff adenocarcinoma (43)	FAP	*APC* c.3927_3931del (p.Glu1309AspfsX4) (29%)
2	A	F	Embryonal Rhabdomyosarcoma (3), Thyroid (18), Peripheral Nerve Sheath Tumor (23), Renal leiomyosarcoma (29), Extraosseous sarcoma (31)	LFS	*TP53* c.743G > A (p.Arg248Gln) (48%)
3	B	F	Breast Cancer (39), Colorectal adenocarcinoma (39), Pleomorphic sarcoma (54), Lung Adenocarcinoma (55)	LFS	*TP53* c.473G > A (p.Arg158His) (57%)
4	C	F	Papillary Thyroid (28),Non-melanotic Skin Cancer (35), Breast Cancer (37), Lung Adenocarcinoma (39)	LFS	*TP53* c.817C > T (p.Arg273Cys) (53%)
5	C	M	Gastro-esophageal junction adenocarcinoma (29)	LFS	*TP53* c.818G > A (p.Arg273His) (52%)
6	C	M	Ileocecal valve adenocarcinoma (36)	LFS	*TP53* c.467G > A (p.Arg156His) (49%)
7	C	M	Esophageal adenocarcinoma (75)	Sch	*SMARCB1* c.143C > T (p.Pro48Leu) (72%)
8	D	F	Gallbladder Cancer (74)	N/A	*TP53* c.524G > A (p.Arg175His) (16%)

*AF* allele frequency, *Cat* category, *F* female, *FAP* familial adenomatous polyposis, *HCS* hereditary cancer syndrome, *LFS* Li-Fraumeni syndrome, *M* male, *Pt* patient, *Sch* schwannomatosis

A variety of distinct scenarios were encountered in patients with gMAVs that were categorized as (Fig. 1):

A. Confirmation of a previously identified cancer predisposition syndrome
B. Identification of a cancer predisposition syndrome in a patient eligible for clinical genetic testing but not previously tested
C. Identification of a potential cancer predisposition syndrome in a patient ineligible for clinical genetic testing
D. A mosaic variant or somatic PBL variant likely not related to an inherited cancer predisposition

For category A patients (Table 2: patients 1 and 2) whose gMAVs were previously identified and disclosed by the clinical genetics service prior to the study, no further action was taken.

One category B patient (Table 2: patient 3) was identified. A woman, who fulfilled Chompret’s Revised criteria for germline *TP53* genetic testing for Li-Fraumeni syndrome (LFS) [15] due to the history of multiple malignancies, however was not referred for a clinical genetics assessment and was found to have a pathogenic variant in *TP53* (c.473G > A; p.Arg158His) consistent with LFS. Unfortunately, the patient died of her malignancy prior to the availability of genetic results and the clinical confirmation could not be performed. Family members were referred for cascade testing on the *TP53* variant identified.

Category C included four patients (Table 2: patients 4–7) who did not meet genetic testing criteria for the identified gMAV at diagnosis of the disease. Patient 4 was a woman who did not meet Chompret’s Revised criteria despite an extensive personal history of cancer. She was enrolled in the tumor profiling program due to a metastatic Her2+ breast cancer and germline DNA analysis revealed a pathogenic variant in *TP53* (c.817C > T; p.Arg273Cys). She presented a prolonged partial response on Her2 therapy and was enrolled in an LFS surveillance program [16] where she was found to have a lung adenocarcinoma. Patient 5, a man diagnosed with a gastro-esophageal junction adenocarcinoma at age 29 years, and Patient 6, a man diagnosed with an ileocecal valve adenocarcinoma at age 36 years, were also found to harbor gMAVs in *TP53*. Interestingly, Patient 6 was found to have a c.467G > A (p.Arg156His) *TP53* germline variant, which in absence of other variants has been reported with conflicting interpretations in ClinVar [17], but when present in conjunction with an additional germline *TP53* variant has been associated with LFS [18]. The tumor analysis of Patient 6 did show another variant in *TP53* (c.742C > T; p.Arg248Trp). Given this potential association with LFS and the tumor results, the Genomic Tumor Board recommended return of this result and further *TP53* analysis to rule out LFS. Sanger sequencing and Multiplex Ligation–dependent Probe Amplification of *TP53* on another sample in a clinical molecular laboratory confirmed and classified the *TP53* c.467G > A variant as a variant of unknown significance, but no other germline variants in *TP53* were identified. Segregation analysis to further characterize the pathogenicity of this variant was not possible due to the unavailability of other family members with cancer history. Patient 7, a 75 year old man with esophageal cancer and no family history of note, was found to have a germline *SMARCB1* variant (c.143C > T; p.Pro48Leu), which has been associated with schwannomatosis and multiple meningiomas [19]. The patient did not have any features of schwannomatosis, but was referred for neurologic assessment and familial testing did not identify the variant in the offspring.

Category D patient (Table 2: patient 8) was a 78 year old adopted woman with a diagnosis of cholangiocarcinoma at age 74, treated with gemcitabine/cisplatin in the metastatic setting prior to her enrollment in the tumor profiling program. PBL DNA analysis found a variant in *TP53* (c.524G > A; p.Arg175His), which has been associated with LFS [20] but was only present at a low allele frequency (16%). Negative cascade testing in the offspring and the absence of variants in *TP53* or other genes in the tumor, suggests that the finding may be due to mosaicism or more plausibly, a treatment related mutation limited to the blood [21, 22]. She declined a skin biopsy for mosaic studies because she was too unwell and shortly after passed away.

## Discussion

Here, we describe the integration of a clinical genetics service in a tumor profiling program not specifically designed to actively seek nor comprehensively analyze germline medically actionable variants (gMAV). Despite this analytical approach of only analyzing gene hotspots, additional gMAVs were found incidentally and disclosed by a clinical genetics service. We also explore patients’ preferences for the return of gMAVs in cancer predisposition genes. We are the first to describe the various scenarios and complexities in incorporating these additional findings into the clinical care of the study patients and families.

Patients expressed great interest in the return of gMAVs (94%), while minority declined (5%) or improperly filled in the consent form (1%). This is consistent with smaller studies such as Gray et al, who reported their experience in 69 lung, colorectal and breast cancer patients, and found that 87% of patients were willing to know about their inherited risk of cancer and 81% of patients agreed to the return of germline information regarding cancer risk and other medically actionable findings [23]. Yusuf et al, reported their experience in 100 breast cancer patients where 90% were willing to know about their cancer risk, while 87% of patients were also interested in other preventable/treatable diseases [24]. In another study from the same group, that included 1167 patients with multiple types of tumors, 99% of the cohort was in agreement to receive information about secondary germline findings [9]. More recently, a study of 413 breast, lung and colorectal cancer patients reported that 77% of patients were interested in germline variants of serious but preventable diseases, while only 56% were interested if the illness was unpreventable. In this study 49% of patients wanted to be informed about variants of unknown significance [25].

Patients’ desire to be informed about additional germline findings remains high over time. Still, the demographic profile of the patients who decline or agree to the return of additional germline results has not been established. In our analysis, there were no statistically significant differences among the two groups in terms of sex, age, race, tumor type and ECOG status. Our cohort was heterogeneous, but with potential bias due to a high number of gynecological (28%) and breast cancer (13%) patients, which enriched the study with predominantly female population (67%).

Advanced cancer patients that are found to harbor previously unrecognized gMAVs in cancer predisposition genes can present multiple challenges for disclosure of the results even if they did consent for the return of additional findings. Alongside with molecular geneticists who determine the pathogenicity of a variant, a critical role is played by the clinical genetics team tasked to disclose germline results to the patient. For this purpose we depict four categories of results (Fig. 1) that highlight the complexity of genetic counseling [26]. Individuals who are eligible for genetic testing are often unrecognized and under-referred [27], and a tumor profiling program may identify a previously eligible patient (Category B) who did not have a genetics assessment. This underscores the importance of enquiring about personal and family cancer history in all cancer patients, especially in those undergoing a tumor molecular profiling that can reveal inherited variants.

On the other hand, current genetic testing criteria, such as Chompret’s Revised criteria for *TP53* genetic testing (patients 4–6) do not capture all the cases and may miss individuals with less striking family histories or de novo cases (Category C). Other disorders such as schwannomatosis (Patient 7) may have low penetrance, making personal and family history unreliable for screening assessment. As exemplified in Patient 4, the identification of atypical hereditary cancer cases provides an opportunity for a patient to undergo appropriate surveillance and the detection of additional malignancies in at risk organs. The widespread use of NGS in tumor profiling programs may complement traditional routes of ascertaining patients and families with a cancer predisposition syndrome.

An emerging area of clinical uncertainty occurs when NGS testing identifies variants in PBL at allele frequency lower than expected for heterozygosity. These low allele frequencies are now detectable using NGS and may be due to a variety of causes such as post-zygotic mosaicism [28], age acquired clonal mosaicism [29, 30] or treatment-related clonal hematopoiesis [22, 28, 31]. This situation was observed in Category D and further follow-up studies are necessary to delineate the etiology of the NGS result, as post-zygotic mosaicism has implications on the family, while age acquired clonal mosaicism and treatment-related clonal hematopoiesis do not.

Our study revealed a total of eight patients with additional gMAVs in a cohort of 1556 advanced cancer patients who underwent NGS tumor profiling. This is likely an under-representation of the true prevalence of hereditary cancer syndromes in our cohort, as this study was not designed to systematically identify all germline genes and variants causing a hereditary cancer syndrome.

Despite these constraints a number of gMAVs were detected as additional findings. As numerous targeted panels perform tumor only sequencing, mostly for economic reasons, these gMAVs may be missed. Our study highlights the potential drawbacks of the tumor-only testing approach since patients were identified with constitutional variants that likely would have been considered somatic with tumor-only NGS panel testing. We also describe the benefit of integrating a tumor profiling program with a clinical genetics service to incorporate these findings into the clinical care of patients. This will ultimately identify more cancer predisposition families and, in turn, preventable cases of cancer.

## Conclusions

Here, we describe the largest cohort reported so far to undergo a precision cancer medicine tumor profiling program, where germline DNA was used primarily to aid in filtering tumour variants. The normal DNA analysis resulted in a variety of returnable additional findings, disclosed through the incorporation of a clinical genetics service within the research study and into the clinical care of these families.

## Additional files

Additional file 1: **Table S1.** 48 genes included in the TruSeq Amplicon Cancer Panel (TSACP, Illumina), which includes 212 amplicons covering a total genomic region 35.84 kb. **Table S2.** 50 genes included in the Ion AmpliSeq Cancer Panel v2 (ASCP, ThermoFisher), which includes 207 amplicons covering a total genomic region of 22 kb. **Table S3.** Cancer Predisposition Genes included on the TruSeq Amplicon Cancer Panel (TSACP) and Ion AmpliSeq Cancer Panel (IACP). Only selected regions (not full exon sequences) of the following genes were included in the panels, which were designed to detect actionable somatic variants. **Table S4.** Patient characteristics. (DOCX 37 kb)

Additional file 2: **Figure S1.** Workflow for additional germline findings. (TIFF 710 kb)

## Abbreviations

ASCP: Ion AmpliSeq Cancer Panel
COMPACT: Community Oncology Molecular Profiling in Advanced Cancers Trial
ECOG: Eastern Cooperative Oncology Group
gMAV: germline medically actionable variant
IGV: Integrative Genomics Viewer
IMPACT: Integrated Molecular Profiling in Advanced Cancers Trial
LFS: Li-Fraumeni syndrome
NGS: Next generation sequencing
PBL: Peripheral blood lymphocytes
TSACP: TruSeq Amplicon Cancer Panel
