# Supplementary material for: Additional germline findings from a tumor profiling program
Source: BMC Med Genomics. 2018 Aug 9;11:65. doi: 10.1186/s12920-018-0383-5 (PMC6085686; doi:10.1186/s12920-018-0383-5)
Supplement: Supplementary file 1 — Table S1. 48 genes included in the TruSeq Amplicon Cancer Panel (TSACP, Illumina), which includes 212 amplicons covering a total genomic region 35.84 kb. Table S2. 50 genes included in the Ion AmpliSeq Cancer Panel v2 (ASCP, ThermoFisher), which includes 207 amplicons covering a total genomic region of 22 kb. Table S3. Cancer Predisposition Genes included on the TruSeq Amplicon Cancer Panel (TSACP) and Ion AmpliSeq Cancer Panel (IACP). Only selected regions (not full exon sequences) of the following genes were included in the panels, which were designed to detect actionable somatic variants. Table S4. Patient characteristics. (DOCX 37 kb) [file 12920_2018_383_MOESM1_ESM.docx]

**Supporting Tables**

**Table S1.** 48 genes included in the TruSeq Amplicon Cancer Panel (TSACP, Illumina), which includes 212 amplicons covering a total genomic region 35.84 kb.

| *ABL1* | ***AKT1*** | ***ALK*** | ***APC* ^†^** |
| --- | --- | --- | --- |
| ***ATM*** | *BRAF* | ***CDH1*** | ***CDKN2A*** |
| *CSF1R* | *CTNNB1* | ***EGFR*** | *ERBB2* |
| *ERBB4* | *FBXW7* | *FGFR1* | *FGFR2* |
| *FGFR3* | *FLT3* | *GNA11* | *GNAS* |
| *GNAQ* | *HNF1A* | *HRAS* | *IDH1* |
| *JAK2* | *JAK3* | *KDR* | ***KIT*** |
| *KRAS* | ***MET*** | ***MLH1* ^†^** | *MPL* |
| *NOTCH1* | *NPM1* | *NRAS* | ***PDGFRA*** |
| ***PIK3CA*** | ***PTEN* ^†^** | *PTPN11* | ***RB1* ^†^** |
| ***RET* ^†^** | ***SMAD4*** | ***SMARCB1*** | *SMO* |
| *SRC* | ***STK11* ^†^** | ***TP53* ^†^** | ***VHL* ^†^** |

In bold: Cancer Predisposition Genes

^†^ American College of Medical Genetics recommendations for reporting of incidental findings in clinical exome and genome sequencing. Genetics in Medicine 2013; 15(7): 565 -547.

**Table S2.** 50 genes included in the Ion AmpliSeq Cancer Panel v2 (ASCP, ThermoFisher), which includes 207 amplicons covering a total genomic region of 22 kb.

| *ABL1* | ***AKT1*** | ***ALK*** | ***APC* ^†^** |
| --- | --- | --- | --- |
| ***ATM*** | *BRAF* | ***CDH1*** | ***CDKN2A*** |
| *CSF1R* | *CTNNB1* | ***EGFR*** | *ERBB2* |
| *ERBB4* | *EZH2* | *FBXW7* | *FGFR1* |
| *FGFR2* | *FGFR3* | *FLT3* | *GNA11* |
| *GNAS* | *GNAQ* | *HNF1A* | *HRAS* |
| *IDH1* | *IDH2* | *JAK2* | *JAK3* |
| *KDR* | ***KIT*** | *KRAS* | ***MET*** |
| ***MLH1* ^†^** | *MPL* | *NOTCH1* | *NPM1* |
| *NRAS* | ***PDGFRA*** | ***PIK3CA*** | ***PTEN* ^†^** |
| *PTPN11* | ***RB1* ^†^** | ***RET* ^†^** | ***SMAD4*** |
| ***SMARCB1*** | *SMO* | *SRC* | ***STK11* ^†^** |
| ***TP53* ^†^** | ***VHL* ^†^** |  |  |

In bold: Cancer Predisposition Genes

^†^ American College of Medical Genetics recommendations for reporting of incidental findings in clinical exome and genome sequencing. Genetics in Medicine 2013; 15(7): 565 -547.

**Table S3:** Cancer Predisposition Genes included on the TruSeq Amplicon Cancer Panel (TSACP) and Ion AmpliSeq Cancer Panel (IACP). Only selected regions (not full exon sequences) of the following genes were included in the panels, which were designed to detect actionable somatic variants.

| Gene | Phenotype | Prevalence Overall Population |
| --- | --- | --- |
| *AKT1* | Cowden Syndrome | 1: 200,000 for Cowden Syndrome |
| *ALK* | Neuroblastoma | UK |
| *APC** | Familial adenomatous polyposis | 2.3:100 000 –3.2:100 000 |
| *ATM^+^* | Ataxia-telangiectasia | 1:40 000 - 1:100 000 |
| *CDH1* | Hereditary diffuse gastric cancer | <0.1: 100 000 |
| *CDKN2A* | Familial melanoma | UK |
| *EGFR* | Non small cell lung cancer | UK |
| *KIT* | Familial GIST | UK |
| *MET* | Hereditary papillary renal cell carcinoma | UK |
| *MLH1** | Lynch syndrome | 1:440 for Lynch Syndrome |
| *PDGRFA* | Familial GIST | UK |
| *PIK3CA* | Cowden Syndrome | 1: 200,000 for Cowden Syndrome |
| *PTEN** | PTEN hamartoma tumor syndrome | 1: 200,000 for Cowden Syndrome |
| *RB1** | Retinoblastoma | 1:15 000 - 1:20 000 |
| *RET** | Multiple endocrine neoplasia type 2 | 1:35 000 |
| *SMAD4* | Juvenile polyposis syndrome | 1:16 000 - 1:100 000 |
| *SMARCB1* | Schwannomatosis/ rhabdoid tumor | 1:33 000 for NF2 |
| *STK11** | Peutz–Jeghers syndrome | 1:25 000 to 1:280 000 |
| *TP53** | Li–Fraumeni syndrome | 1:5 000 - 1:20 000 |
| *VHL** | Von Hippel–Lindau syndrome | 1:36 000 |

* American College of Medical Genetics and Genomics (ACMG) recommendations for reporting of incidental findings in clinical exome and genome sequencing. Genetics in Medicine 2013; 15(7): 565 -547. ***^+^*** Estimated frequency of homozygotic ATM germline variant linked to ataxia-telangiectasia; GIST: Gastrointestinal stromal tumors; Sdr: Syndrome. NF2: Neurofibromatosis type 2: UK: Unknown.

## Table S4: Patient characteristics

| Characteristic | Value |
| --- | --- |
|  | **median (range)** |
| Age | 58 (18-89) |
|  | **n (%)** |
| Sex |  |
| - Male | 646 (33%) |
| - Female | 1314 (67%) |
| Race |  |
| - White | 1067 (54%) |
| - Black | 36 (2%) |
| - Asian | 225 (12%) |
| - Mixed | 14 (1%) |
| - UK | 618 (31%) |
| Disease Site |  |
| - Gynecological | 548 (28%) |
| - Gastro-Intestinal | 403 (21%) |
| - Breast | 253 (13%) |
| - Lung | 244 (12%) |
| - Pancreatobiliary | 203 (10%) |
| - Genito-Urinary | 98 (5%) |
| - Rare Tumors | 41 (2%) |
| - Head and Neck | 40 (2%) |
| - Melanoma | 38 (2%) |
| - Thyroid | 33 (2%) |
| - Unknown Origin | 33 (2%) |
| - Sarcoma | 26 (1%) |
| ECOG |  |
| - 0 | 802 (41%) |
| - 1 | 1158 (59%) |
| Prior Genetic Testing |  |
| - Yes | 361 (18%) |
| - No | 1599 (82%) |

ECOG: Eastern Cooperative Oncology Group; n: number; UK: Unknown.
