# Supplementary figures and images for: Additional germline findings from a tumor profiling program
Source: BMC Med Genomics. 2018 Aug 9;11:65. doi: 10.1186/s12920-018-0383-5 (PMC6085686; doi:10.1186/s12920-018-0383-5)

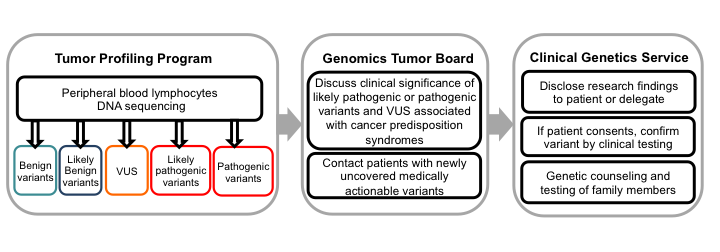

Supplement: Supplementary file 2 — Figure S1. Workflow for additional germline findings. (TIFF 710 kb) [file 12920_2018_383_MOESM2_ESM.tiff]
